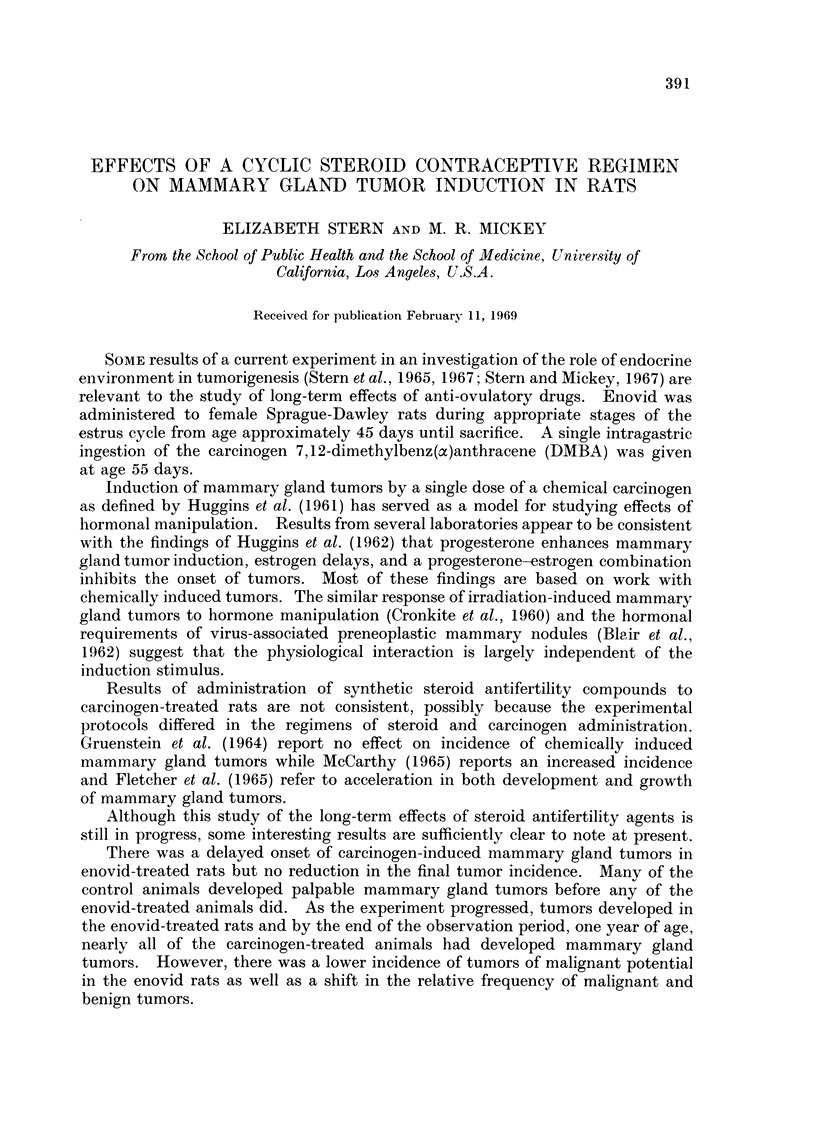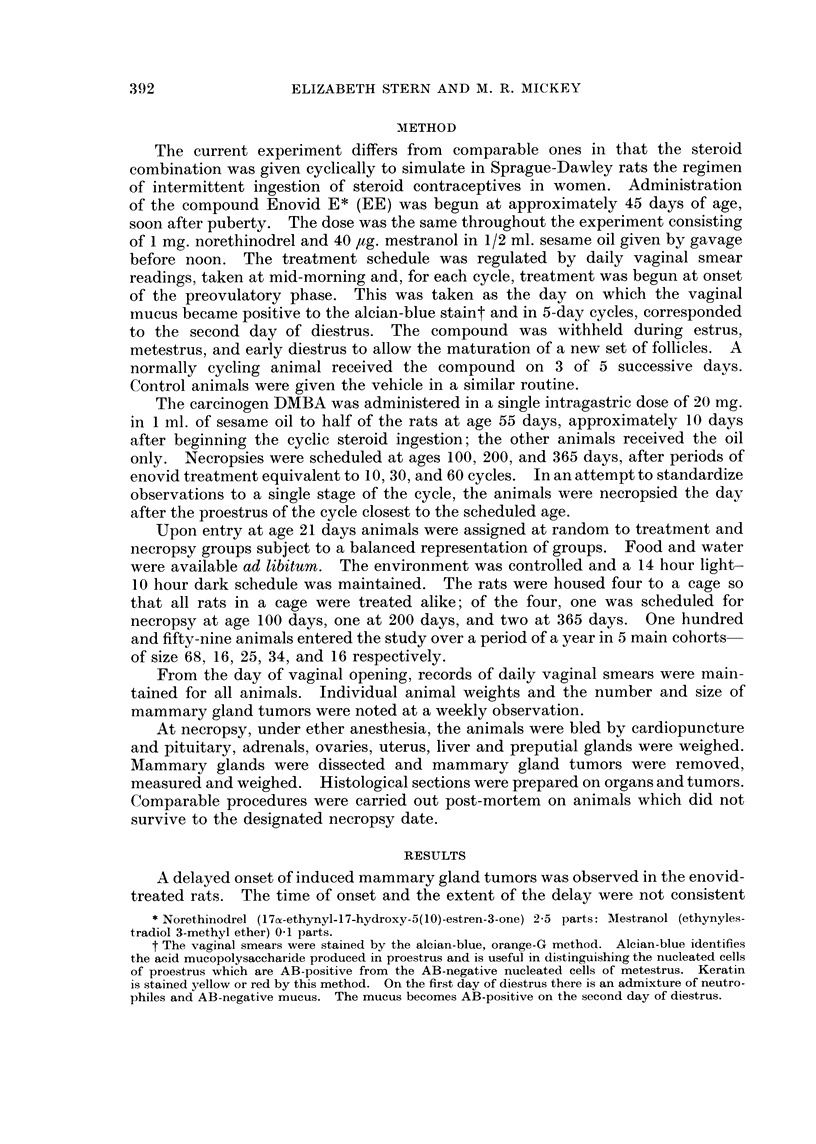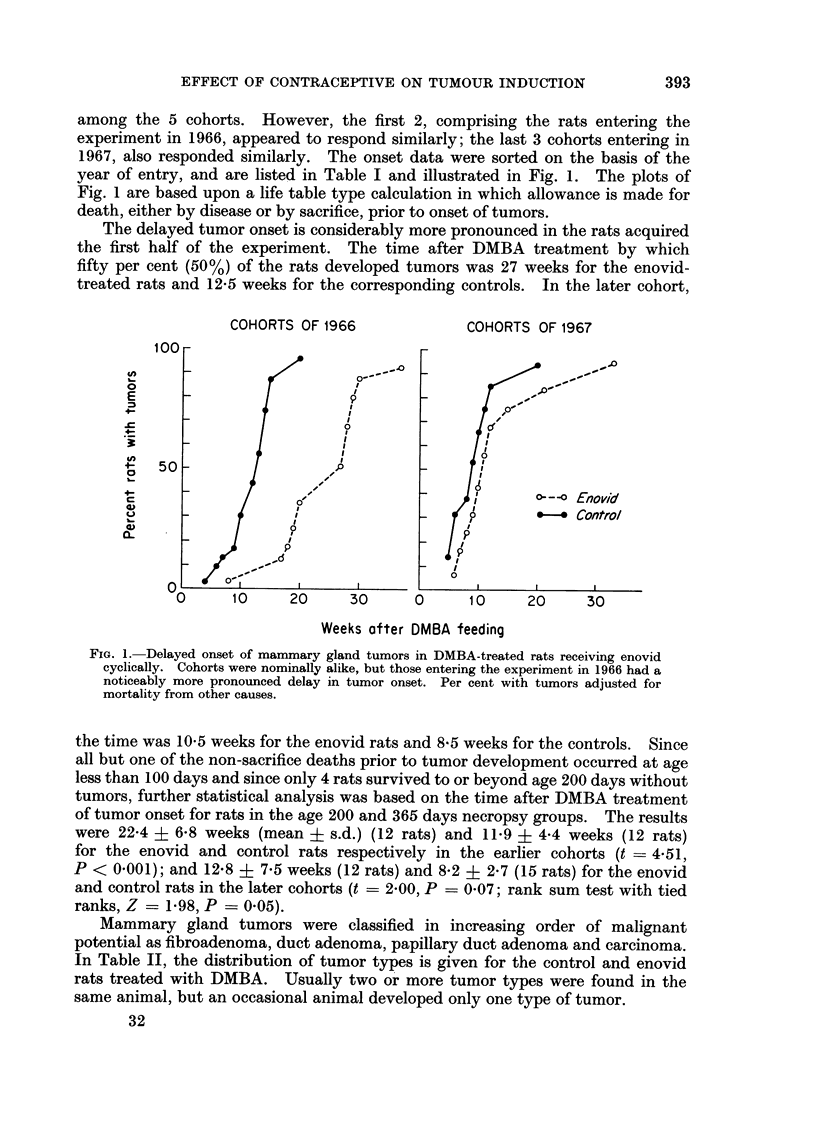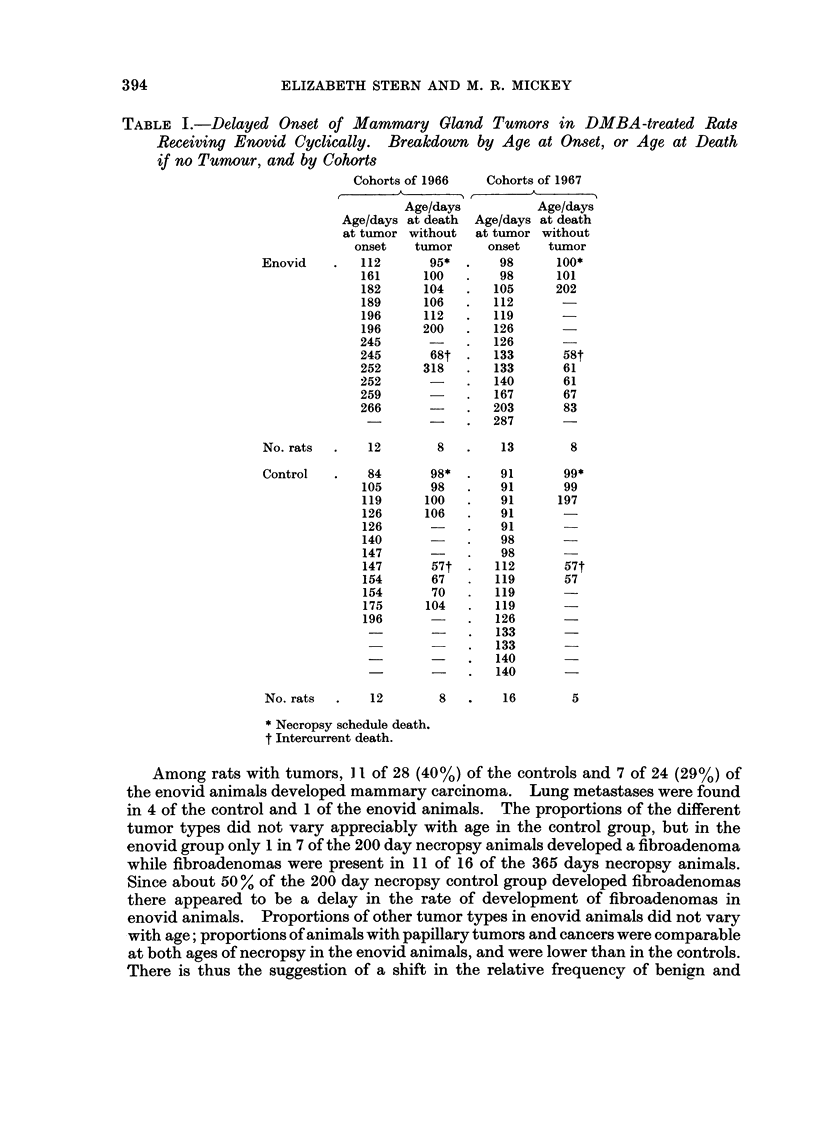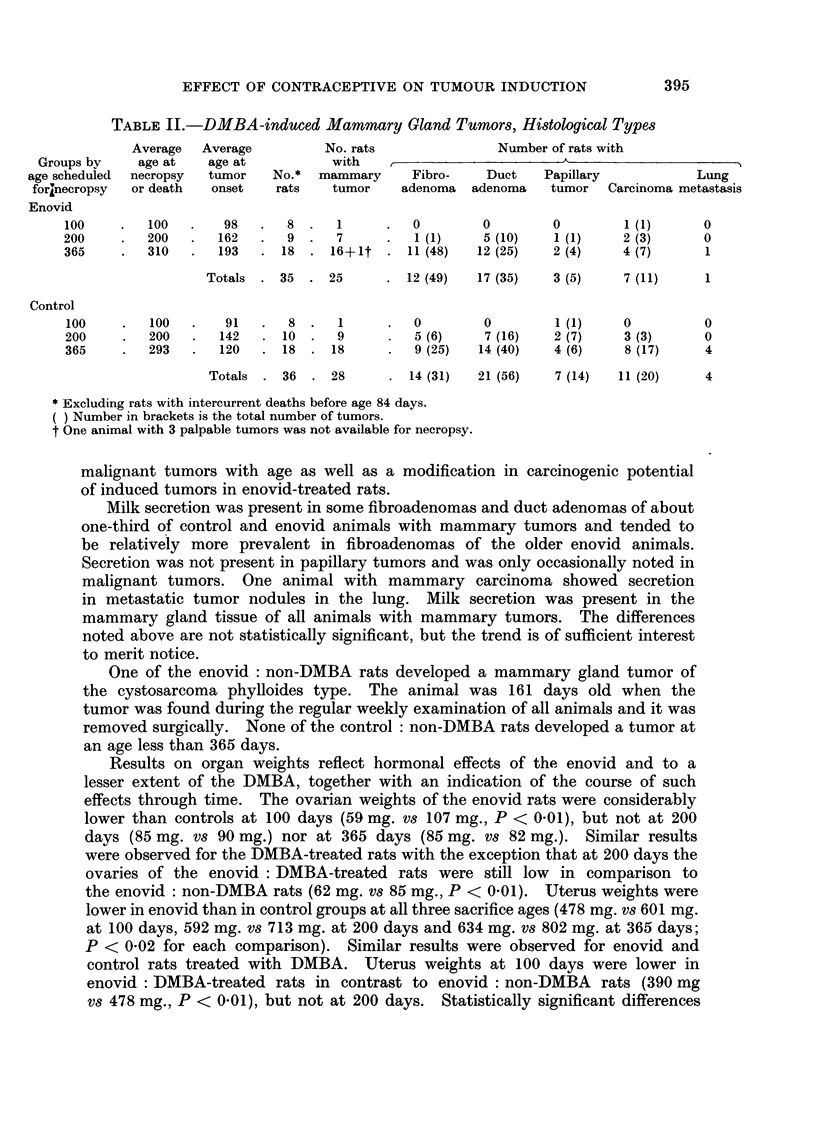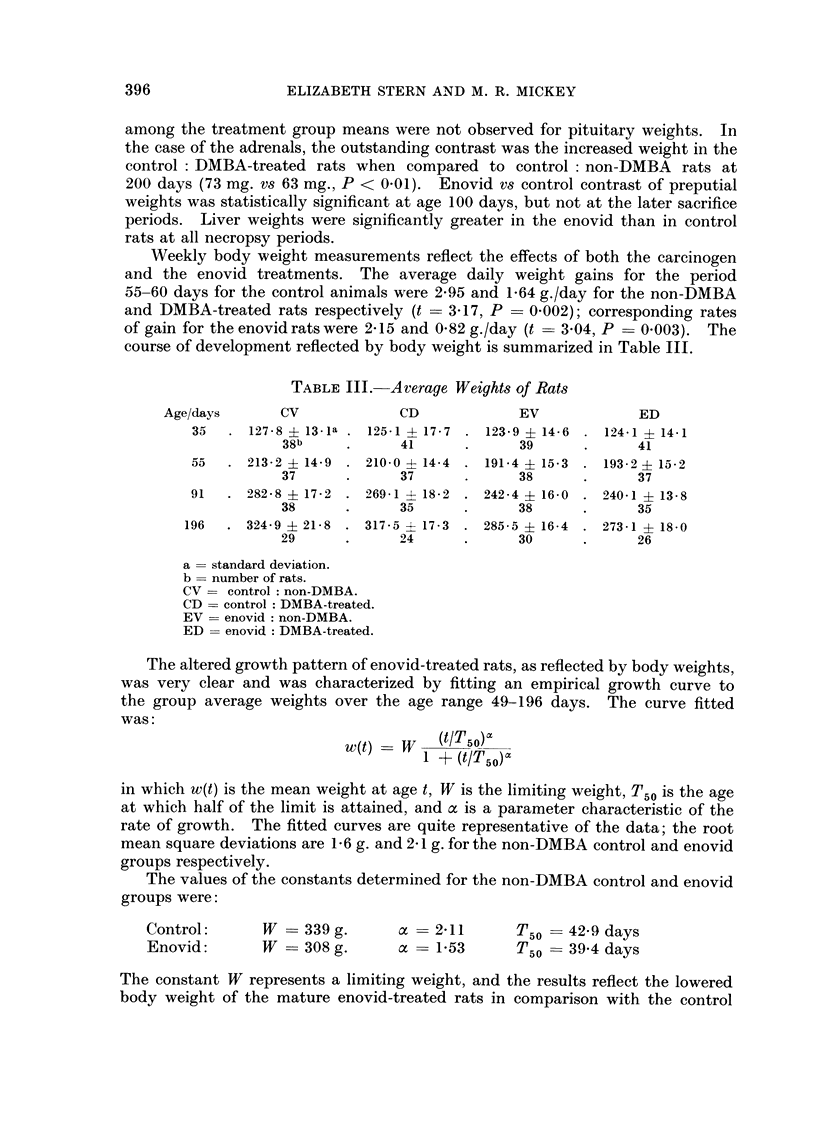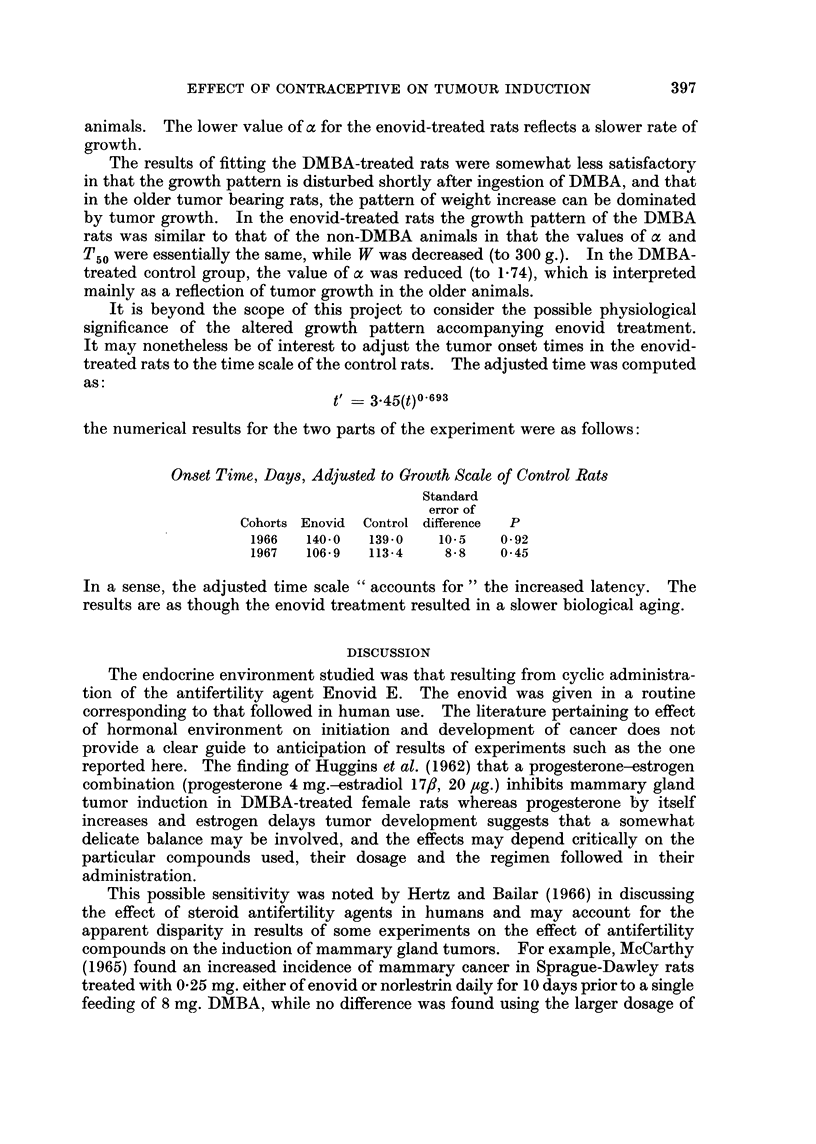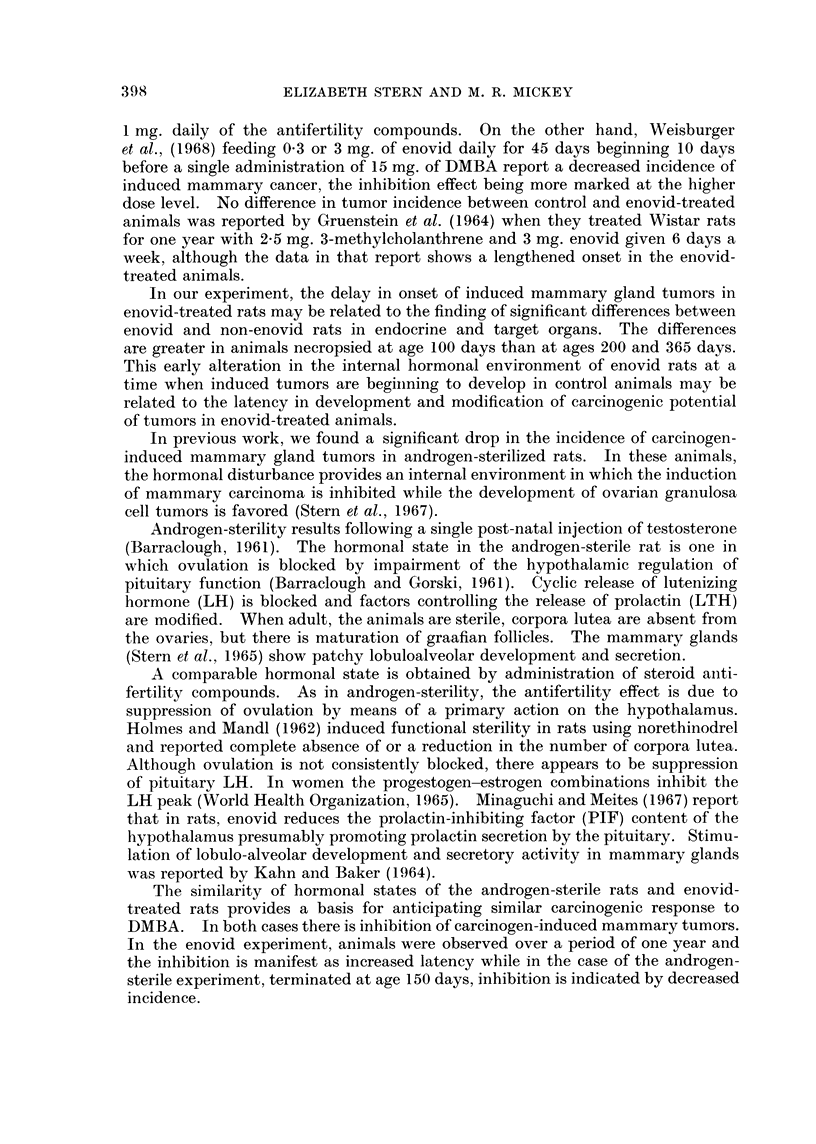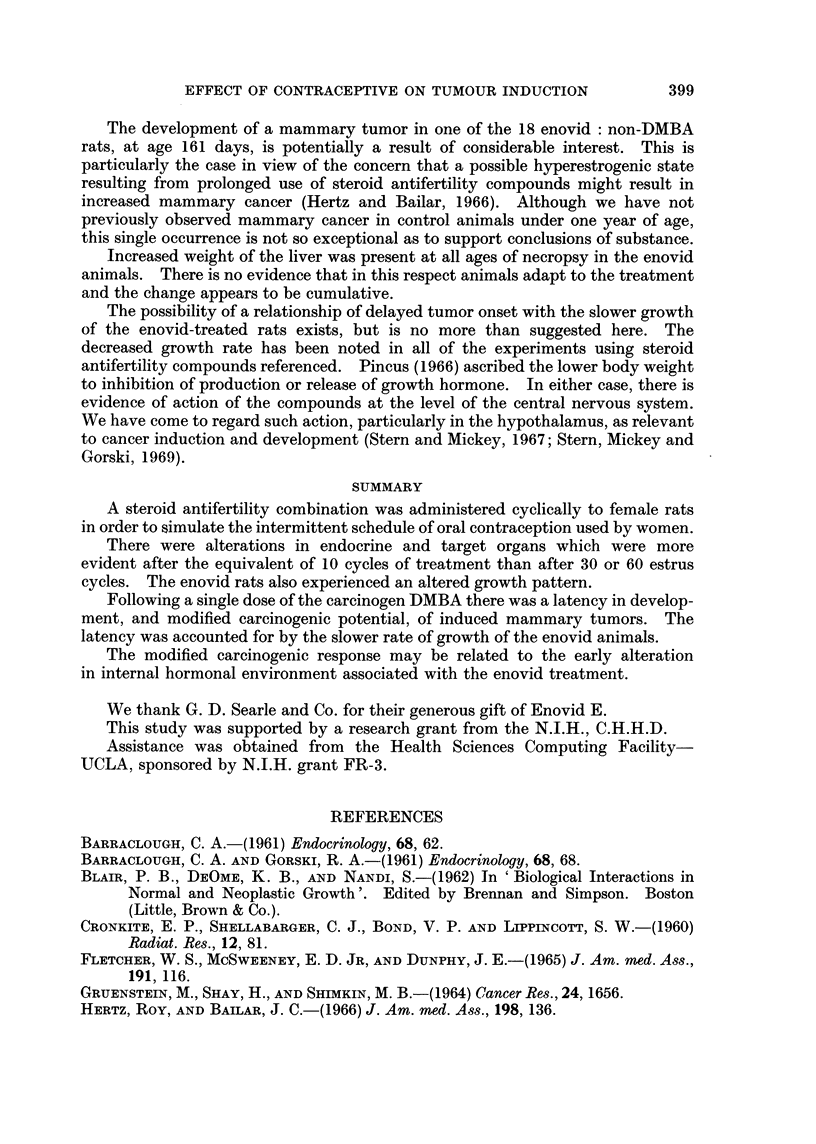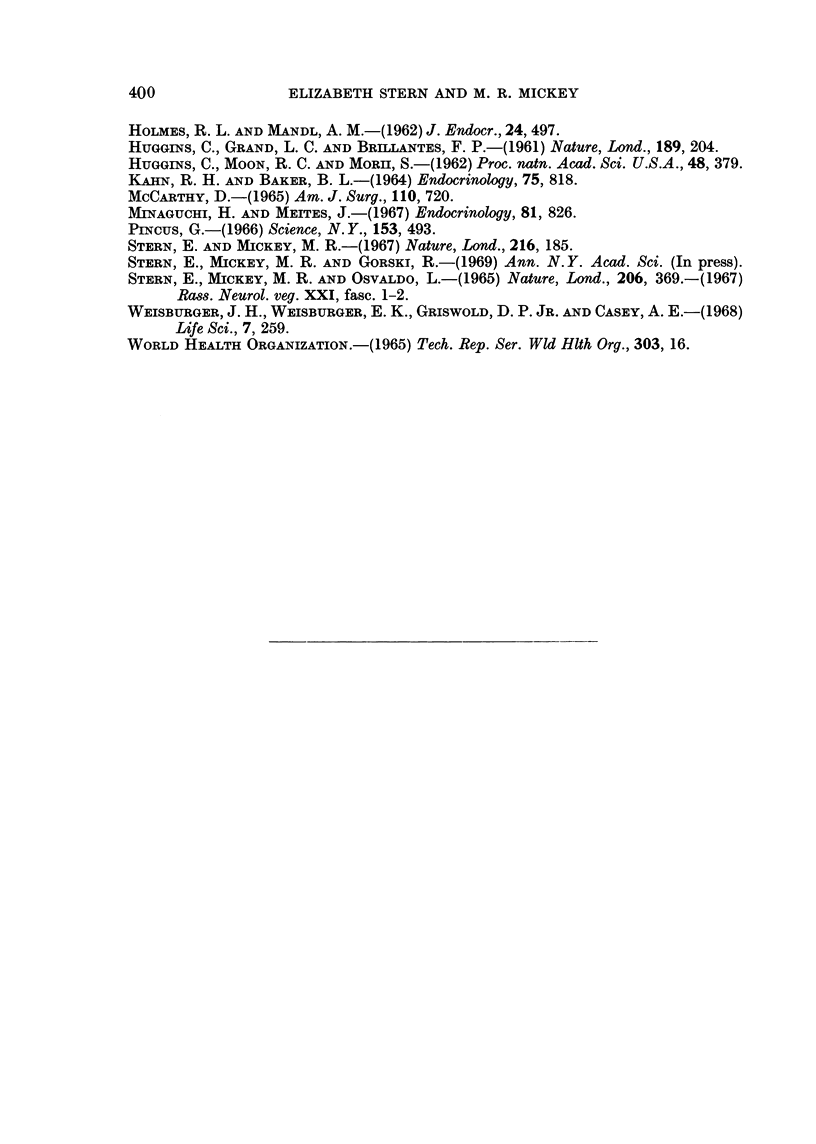# Effects of a cyclic steroid contraceptive regimen on mammary gland tumor induction in rats.

**DOI:** 10.1038/bjc.1969.51

**Published:** 1969-06

**Authors:** E. Stern, M. R. Mickey


### EFFECTS OF A CYCLIC STEROID CONTRACEPTIVE REGIMEN ON MAMMARY GLAND TUMOR INDUCTION IN RATS

ELIZABETH STERN AND M. R. MICKEY

*From the School of Public Health and the School of Medicine, University of California, Los Angeles, U.S.A.*

Received for publication February 11, 1969

SOME results of a current experiment in an investigation of the role of endocrine environment in tumorigenesis (Stern *et al.*, 1965, 1967; Stern and Mickey, 1967) are relevant to the study of long-term effects of anti-ovulatory drugs. Enovid was administered to female Sprague-Dawley rats during appropriate stages of the estrus cycle from age approximately 45 days until sacrifice. A single intragastric ingestion of the carcinogen 7,12-dimethylbenz($\alpha$)anthracene (DMBA) was given at age 55 days.

Induction of mammary gland tumors by a single dose of a chemical carcinogen as defined by Huggins *et al.* (1961) has served as a model for studying effects of hormonal manipulation. Results from several laboratories appear to be consistent with the findings of Huggins *et al.* (1962) that progesterone enhances mammary gland tumor induction, estrogen delays, and a progesterone–estrogen combination inhibits the onset of tumors. Most of these findings are based on work with chemically induced tumors. The similar response of irradiation-induced mammary gland tumors to hormone manipulation (Cronkite *et al.*, 1960) and the hormonal requirements of virus-associated preneoplastic mammary nodules (Blair *et al.*, 1962) suggest that the physiological interaction is largely independent of the induction stimulus.

Results of administration of synthetic steroid antifertility compounds to carcinogen-treated rats are not consistent, possibly because the experimental protocols differed in the regimens of steroid and carcinogen administration. Gruenstein *et al.* (1964) report no effect on incidence of chemically induced mammary gland tumors while McCarthy (1965) reports an increased incidence and Fletcher *et al.* (1965) refer to acceleration in both development and growth of mammary gland tumors.

Although this study of the long-term effects of steroid antifertility agents is still in progress, some interesting results are sufficiently clear to note at present.

There was a delayed onset of carcinogen-induced mammary gland tumors in enovid-treated rats but no reduction in the final tumor incidence. Many of the control animals developed palpable mammary gland tumors before any of the enovid-treated animals did. As the experiment progressed, tumors developed in the enovid-treated rats and by the end of the observation period, one year of age, nearly all of the carcinogen-treated animals had developed mammary gland tumors. However, there was a lower incidence of tumors of malignant potential in the enovid rats as well as a shift in the relative frequency of malignant and benign tumors.

### METHOD

The current experiment differs from comparable ones in that the steroid combination was given cyclically to simulate in Sprague-Dawley rats the regimen of intermittent ingestion of steroid contraceptives in women. Administration of the compound Enovid E* (EE) was begun at approximately 45 days of age, soon after puberty. The dose was the same throughout the experiment consisting of 1 mg. norethinodrel and 40 $\mu$g. mestranol in 1/2 ml. sesame oil given by gavage before noon. The treatment schedule was regulated by daily vaginal smear readings, taken at mid-morning and, for each cycle, treatment was begun at onset of the preovulatory phase. This was taken as the day on which the vaginal mucus became positive to the alcian-blue stain† and in 5-day cycles, corresponded to the second day of diestrus. The compound was withheld during estrus, metestrus, and early diestrus to allow the maturation of a new set of follicles. A normally cycling animal received the compound on 3 of 5 successive days. Control animals were given the vehicle in a similar routine.

The carcinogen DMBA was administered in a single intragastric dose of 20 mg. in 1 ml. of sesame oil to half of the rats at age 55 days, approximately 10 days after beginning the cyclic steroid ingestion; the other animals received the oil only. Necropsies were scheduled at ages 100, 200, and 365 days, after periods of enovid treatment equivalent to 10, 30, and 60 cycles. In an attempt to standardize observations to a single stage of the cycle, the animals were necropsied the day after the proestrus of the cycle closest to the scheduled age.

Upon entry at age 21 days animals were assigned at random to treatment and necropsy groups subject to a balanced representation of groups. Food and water were available *ad libitum.* The environment was controlled and a 14 hour light–10 hour dark schedule was maintained. The rats were housed four to a cage so that all rats in a cage were treated alike; of the four, one was scheduled for necropsy at age 100 days, one at 200 days, and two at 365 days. One hundred and fifty-nine animals entered the study over a period of a year in 5 main cohorts—of size 68, 16, 25, 34, and 16 respectively.

From the day of vaginal opening, records of daily vaginal smears were maintained for all animals. Individual animal weights and the number and size of mammary gland tumors were noted at a weekly observation.

At necropsy, under ether anesthesia, the animals were bled by cardiopuncture and pituitary, adrenals, ovaries, uterus, liver and preputial glands were weighed. Mammary glands were dissected and mammary gland tumors were removed, measured and weighed. Histological sections were prepared on organs and tumors. Comparable procedures were carried out post-mortem on animals which did not survive to the designated necropsy date.

### RESULTS

A delayed onset of induced mammary gland tumors was observed in the enovid-treated rats. The time of onset and the extent of the delay were not consistent

* Norethinodrel (17$\alpha$-ethynyl-17-hydroxy-5(10)-estren-3-one) 2·5 parts: Mestranol (ethynylestradiol 3-methyl ether) 0·1 parts.

† The vaginal smears were stained by the alcian-blue, orange-G method. Alcian-blue identifies the acid mucopolysaccharide produced in proestrus and is useful in distinguishing the nucleated cells of proestrus which are AB-positive from the AB-negative nucleated cells of metestrus. Keratin is stained yellow or red by this method. On the first day of diestrus there is an admixture of neutrophiles and AB-negative mucus. The mucus becomes AB-positive on the second day of diestrus.

among the 5 cohorts. However, the first 2, comprising the rats entering the experiment in 1966, appeared to respond similarly; the last 3 cohorts entering in 1967, also responded similarly. The onset data were sorted on the basis of the year of entry, and are listed in Table I and illustrated in Fig. 1. The plots of Fig. 1 are based upon a life table type calculation in which allowance is made for death, either by disease or by sacrifice, prior to onset of tumors.

The delayed tumor onset is considerably more pronounced in the rats acquired the first half of the experiment. The time after DMBA treatment by which fifty per cent (50%) of the rats developed tumors was 27 weeks for the enovid-treated rats and 12·5 weeks for the corresponding controls. In the later cohort,

FIG. 1.—Delayed onset of mammary gland tumors in DMBA-treated rats receiving enovid cyclically. Cohorts were nominally alike, but those entering the experiment in 1966 had a noticeably more pronounced delay in tumor onset. Per cent with tumors adjusted for mortality from other causes.

the time was 10·5 weeks for the enovid rats and 8·5 weeks for the controls. Since all but one of the non-sacrifice deaths prior to tumor development occurred at age less than 100 days and since only 4 rats survived to or beyond age 200 days without tumors, further statistical analysis was based on the time after DMBA treatment of tumor onset for rats in the age 200 and 365 days necropsy groups. The results were 22·4 $\pm$ 6·8 weeks (mean $\pm$ s.d.) (12 rats) and 11·9 $\pm$ 4·4 weeks (12 rats) for the enovid and control rats respectively in the earlier cohorts ($t = 4{\cdot}51$, $P < 0{\cdot}001$); and 12·8 $\pm$ 7·5 weeks (12 rats) and 8·2 $\pm$ 2·7 (15 rats) for the enovid and control rats in the later cohorts ($t = 2{\cdot}00$, $P = 0{\cdot}07$; rank sum test with tied ranks, $Z = 1{\cdot}98$, $P = 0{\cdot}05$).

Mammary gland tumors were classified in increasing order of malignant potential as fibroadenoma, duct adenoma, papillary duct adenoma and carcinoma. In Table II, the distribution of tumor types is given for the control and enovid rats treated with DMBA. Usually two or more tumor types were found in the same animal, but an occasional animal developed only one type of tumor.

TABLE I.—*Delayed Onset of Mammary Gland Tumors in DMBA-treated Rats Receiving Enovid Cyclically. Breakdown by Age at Onset, or Age at Death if no Tumour, and by Cohorts*

| | Cohorts of 1966 | | Cohorts of 1967 | |
|---|---|---|---|---|
| | Age/days at tumor onset | Age/days at death without tumor | Age/days at tumor onset | Age/days at death without tumor |
| Enovid | 112 | 95* | 98 | 100* |
| | 161 | 100 | 98 | 101 |
| | 182 | 104 | 105 | 202 |
| | 189 | 106 | 112 | — |
| | 196 | 112 | 119 | — |
| | 196 | 200 | 126 | — |
| | 245 | — | 126 | — |
| | 245 | 68† | 133 | 58† |
| | 252 | 318 | 133 | 61 |
| | 252 | — | 140 | 61 |
| | 259 | — | 167 | 67 |
| | 266 | — | 203 | 83 |
| | — | — | 287 | — |
| No. rats | 12 | 8 | 13 | 8 |
| Control | 84 | 98* | 91 | 99* |
| | 105 | 98 | 91 | 99 |
| | 119 | 100 | 91 | 197 |
| | 126 | 106 | 91 | — |
| | 126 | — | 91 | — |
| | 140 | — | 98 | — |
| | 147 | — | 98 | — |
| | 147 | 57† | 112 | 57† |
| | 154 | 67 | 119 | 57 |
| | 154 | 70 | 119 | — |
| | 175 | 104 | 119 | — |
| | 196 | — | 126 | — |
| | — | — | 133 | — |
| | — | — | 133 | — |
| | — | — | 140 | — |
| | — | — | 140 | — |
| No. rats | 12 | 8 | 16 | 5 |

* Necropsy schedule death.
† Intercurrent death.

Among rats with tumors, 11 of 28 (40%) of the controls and 7 of 24 (29%) of the enovid animals developed mammary carcinoma. Lung metastases were found in 4 of the control and 1 of the enovid animals. The proportions of the different tumor types did not vary appreciably with age in the control group, but in the enovid group only 1 in 7 of the 200 day necropsy animals developed a fibroadenoma while fibroadenomas were present in 11 of 16 of the 365 days necropsy animals. Since about 50% of the 200 day necropsy control group developed fibroadenomas there appeared to be a delay in the rate of development of fibroadenomas in enovid animals. Proportions of other tumor types in enovid animals did not vary with age; proportions of animals with papillary tumors and cancers were comparable at both ages of necropsy in the enovid animals, and were lower than in the controls. There is thus the suggestion of a shift in the relative frequency of benign and

TABLE II.—*DMBA-induced Mammary Gland Tumors, Histological Types*

| Groups by age scheduled for necropsy | Average age at necropsy or death | Average age at tumor onset | No.* rats | No. rats with mammary tumor | Number of rats with: Fibro-adenoma | Duct adenoma | Papillary tumor | Carcinoma | Lung metastasis |
|---|---|---|---|---|---|---|---|---|---|
| Enovid | | | | | | | | | |
| 100 | 100 | 98 | 8 | 1 | 0 | 0 | 0 | 1 (1) | 0 |
| 200 | 200 | 162 | 9 | 7 | 1 (1) | 5 (10) | 1 (1) | 2 (3) | 0 |
| 365 | 310 | 193 | 18 | 16+1† | 11 (48) | 12 (25) | 2 (4) | 4 (7) | 1 |
| | | Totals | 35 | 25 | 12 (49) | 17 (35) | 3 (5) | 7 (11) | 1 |
| Control | | | | | | | | | |
| 100 | 100 | 91 | 8 | 1 | 0 | 0 | 1 (1) | 0 | 0 |
| 200 | 200 | 142 | 10 | 9 | 5 (6) | 7 (16) | 2 (7) | 3 (3) | 0 |
| 365 | 293 | 120 | 18 | 18 | 9 (25) | 14 (40) | 4 (6) | 8 (17) | 4 |
| | | Totals | 36 | 28 | 14 (31) | 21 (56) | 7 (14) | 11 (20) | 4 |

* Excluding rats with intercurrent deaths before age 84 days.
( ) Number in brackets is the total number of tumors.
† One animal with 3 palpable tumors was not available for necropsy.

malignant tumors with age as well as a modification in carcinogenic potential of induced tumors in enovid-treated rats.

Milk secretion was present in some fibroadenomas and duct adenomas of about one-third of control and enovid animals with mammary tumors and tended to be relatively more prevalent in fibroadenomas of the older enovid animals. Secretion was not present in papillary tumors and was only occasionally noted in malignant tumors. One animal with mammary carcinoma showed secretion in metastatic tumor nodules in the lung. Milk secretion was present in the mammary gland tissue of all animals with mammary tumors. The differences noted above are not statistically significant, but the trend is of sufficient interest to merit notice.

One of the enovid : non-DMBA rats developed a mammary gland tumor of the cystosarcoma phylloides type. The animal was 161 days old when the tumor was found during the regular weekly examination of all animals and it was removed surgically. None of the control : non-DMBA rats developed a tumor at an age less than 365 days.

Results on organ weights reflect hormonal effects of the enovid and to a lesser extent of the DMBA, together with an indication of the course of such effects through time. The ovarian weights of the enovid rats were considerably lower than controls at 100 days (59 mg. *vs* 107 mg., $P < 0{\cdot}01$), but not at 200 days (85 mg. *vs* 90 mg.) nor at 365 days (85 mg. *vs* 82 mg.). Similar results were observed for the DMBA-treated rats with the exception that at 200 days the ovaries of the enovid : DMBA-treated rats were still low in comparison to the enovid : non-DMBA rats (62 mg. *vs* 85 mg., $P < 0{\cdot}01$). Uterus weights were lower in enovid than in control groups at all three sacrifice ages (478 mg. *vs* 601 mg. at 100 days, 592 mg. *vs* 713 mg. at 200 days and 634 mg. *vs* 802 mg. at 365 days; $P < 0{\cdot}02$ for each comparison). Similar results were observed for enovid and control rats treated with DMBA. Uterus weights at 100 days were lower in enovid : DMBA-treated rats in contrast to enovid : non-DMBA rats (390 mg *vs* 478 mg., $P < 0{\cdot}01$), but not at 200 days. Statistically significant differences among the treatment group means were not observed for pituitary weights. In the case of the adrenals, the outstanding contrast was the increased weight in the control : DMBA-treated rats when compared to control : non-DMBA rats at 200 days (73 mg. *vs* 63 mg., $P < 0{\cdot}01$). Enovid *vs* control contrast of preputial weights was statistically significant at age 100 days, but not at the later sacrifice periods. Liver weights were significantly greater in the enovid than in control rats at all necropsy periods.

Weekly body weight measurements reflect the effects of both the carcinogen and the enovid treatments. The average daily weight gains for the period 55–60 days for the control animals were 2·95 and 1·64 g./day for the non-DMBA and DMBA-treated rats respectively ($t = 3{\cdot}17$, $P = 0{\cdot}002$); corresponding rates of gain for the enovid rats were 2·15 and 0·82 g./day ($t = 3{\cdot}04$, $P = 0{\cdot}003$). The course of development reflected by body weight is summarized in Table III.

TABLE III.—*Average Weights of Rats*

| Age/days | CV | CD | EV | ED |
|---|---|---|---|---|
| 35 | 127·8 ± 13·1[a] | 125·1 ± 17·7 | 123·9 ± 14·6 | 124·1 ± 14·1 |
| | 38[b] | 41 | 39 | 41 |
| 55 | 213·2 ± 14·9 | 210·0 ± 14·4 | 191·4 ± 15·3 | 193·2 ± 15·2 |
| | 37 | 37 | 38 | 37 |
| 91 | 282·8 ± 17·2 | 269·1 ± 18·2 | 242·4 ± 16·0 | 240·1 ± 13·8 |
| | 38 | 35 | 38 | 35 |
| 196 | 324·9 ± 21·8 | 317·5 ± 17·3 | 285·5 ± 16·4 | 273·1 ± 18·0 |
| | 29 | 24 | 30 | 26 |

a = standard deviation.
b = number of rats.
CV = control : non-DMBA.
CD = control : DMBA-treated.
EV = enovid : non-DMBA.
ED = enovid : DMBA-treated.

The altered growth pattern of enovid-treated rats, as reflected by body weights, was very clear and was characterized by fitting an empirical growth curve to the group average weights over the age range 49–196 days. The curve fitted was:

$$w(t) = W\frac{(t/T_{50})^{\alpha}}{1 + (t/T_{50})^{\alpha}}$$

in which $w(t)$ is the mean weight at age $t$, $W$ is the limiting weight, $T_{50}$ is the age at which half of the limit is attained, and $\alpha$ is a parameter characteristic of the rate of growth. The fitted curves are quite representative of the data; the root mean square deviations are 1·6 g. and 2·1 g. for the non-DMBA control and enovid groups respectively.

The values of the constants determined for the non-DMBA control and enovid groups were:

| | | | |
|---|---|---|---|
| Control: | $W = 339$ g. | $\alpha = 2{\cdot}11$ | $T_{50} = 42{\cdot}9$ days |
| Enovid: | $W = 308$ g. | $\alpha = 1{\cdot}53$ | $T_{50} = 39{\cdot}4$ days |

The constant $W$ represents a limiting weight, and the results reflect the lowered body weight of the mature enovid-treated rats in comparison with the control animals. The lower value of $\alpha$ for the enovid-treated rats reflects a slower rate of growth.

The results of fitting the DMBA-treated rats were somewhat less satisfactory in that the growth pattern is disturbed shortly after ingestion of DMBA, and that in the older tumor bearing rats, the pattern of weight increase can be dominated by tumor growth. In the enovid-treated rats the growth pattern of the DMBA rats was similar to that of the non-DMBA animals in that the values of $\alpha$ and $T_{50}$ were essentially the same, while $W$ was decreased (to 300 g.). In the DMBA-treated control group, the value of $\alpha$ was reduced (to 1·74), which is interpreted mainly as a reflection of tumor growth in the older animals.

It is beyond the scope of this project to consider the possible physiological significance of the altered growth pattern accompanying enovid treatment. It may nonetheless be of interest to adjust the tumor onset times in the enovid-treated rats to the time scale of the control rats. The adjusted time was computed as:

$$t' = 3{\cdot}45(t)^{0{\cdot}693}$$

the numerical results for the two parts of the experiment were as follows:

*Onset Time, Days, Adjusted to Growth Scale of Control Rats*

| Cohorts | Enovid | Control | Standard error of difference | *P* |
|---|---|---|---|---|
| 1966 | 140·0 | 139·0 | 10·5 | 0·92 |
| 1967 | 106·9 | 113·4 | 8·8 | 0·45 |

In a sense, the adjusted time scale " accounts for " the increased latency. The results are as though the enovid treatment resulted in a slower biological aging.

### DISCUSSION

The endocrine environment studied was that resulting from cyclic administration of the antifertility agent Enovid E. The enovid was given in a routine corresponding to that followed in human use. The literature pertaining to effect of hormonal environment on initiation and development of cancer does not provide a clear guide to anticipation of results of experiments such as the one reported here. The finding of Huggins *et al.* (1962) that a progesterone–estrogen combination (progesterone 4 mg.–estradiol 17$\beta$, 20 $\mu$g.) inhibits mammary gland tumor induction in DMBA-treated female rats whereas progesterone by itself increases and estrogen delays tumor development suggests that a somewhat delicate balance may be involved, and the effects may depend critically on the particular compounds used, their dosage and the regimen followed in their administration.

This possible sensitivity was noted by Hertz and Bailar (1966) in discussing the effect of steroid antifertility agents in humans and may account for the apparent disparity in results of some experiments on the effect of antifertility compounds on the induction of mammary gland tumors. For example, McCarthy (1965) found an increased incidence of mammary cancer in Sprague-Dawley rats treated with 0·25 mg. either of enovid or norlestrin daily for 10 days prior to a single feeding of 8 mg. DMBA, while no difference was found using the larger dosage of

1 mg. daily of the antifertility compounds. On the other hand, Weisburger *et al.*, (1968) feeding 0·3 or 3 mg. of enovid daily for 45 days beginning 10 days before a single administration of 15 mg. of DMBA report a decreased incidence of induced mammary cancer, the inhibition effect being more marked at the higher dose level. No difference in tumor incidence between control and enovid-treated animals was reported by Gruenstein *et al.* (1964) when they treated Wistar rats for one year with 2·5 mg. 3-methylcholanthrene and 3 mg. enovid given 6 days a week, although the data in that report shows a lengthened onset in the enovid-treated animals.

In our experiment, the delay in onset of induced mammary gland tumors in enovid-treated rats may be related to the finding of significant differences between enovid and non-enovid rats in endocrine and target organs. The differences are greater in animals necropsied at age 100 days than at ages 200 and 365 days. This early alteration in the internal hormonal environment of enovid rats at a time when induced tumors are beginning to develop in control animals may be related to the latency in development and modification of carcinogenic potential of tumors in enovid-treated animals.

In previous work, we found a significant drop in the incidence of carcinogen-induced mammary gland tumors in androgen-sterilized rats. In these animals, the hormonal disturbance provides an internal environment in which the induction of mammary carcinoma is inhibited while the development of ovarian granulosa cell tumors is favored (Stern *et al.*, 1967).

Androgen-sterility results following a single post-natal injection of testosterone (Barraclough, 1961). The hormonal state in the androgen-sterile rat is one in which ovulation is blocked by impairment of the hypothalamic regulation of pituitary function (Barraclough and Gorski, 1961). Cyclic release of lutenizing hormone (LH) is blocked and factors controlling the release of prolactin (LTH) are modified. When adult, the animals are sterile, corpora lutea are absent from the ovaries, but there is maturation of graafian follicles. The mammary glands (Stern *et al.*, 1965) show patchy lobuloalveolar development and secretion.

A comparable hormonal state is obtained by administration of steroid antifertility compounds. As in androgen-sterility, the antifertility effect is due to suppression of ovulation by means of a primary action on the hypothalamus. Holmes and Mandl (1962) induced functional sterility in rats using norethinodrel and reported complete absence of or a reduction in the number of corpora lutea. Although ovulation is not consistently blocked, there appears to be suppression of pituitary LH. In women the progestogen–estrogen combinations inhibit the LH peak (World Health Organization, 1965). Minaguchi and Meites (1967) report that in rats, enovid reduces the prolactin-inhibiting factor (PIF) content of the hypothalamus presumably promoting prolactin secretion by the pituitary. Stimulation of lobulo-alveolar development and secretory activity in mammary glands was reported by Kahn and Baker (1964).

The similarity of hormonal states of the androgen-sterile rats and enovid-treated rats provides a basis for anticipating similar carcinogenic response to DMBA. In both cases there is inhibition of carcinogen-induced mammary tumors. In the enovid experiment, animals were observed over a period of one year and the inhibition is manifest as increased latency while in the case of the androgen-sterile experiment, terminated at age 150 days, inhibition is indicated by decreased incidence.

The development of a mammary tumor in one of the 18 enovid : non-DMBA rats, at age 161 days, is potentially a result of considerable interest. This is particularly the case in view of the concern that a possible hyperestrogenic state resulting from prolonged use of steroid antifertility compounds might result in increased mammary cancer (Hertz and Bailar, 1966). Although we have not previously observed mammary cancer in control animals under one year of age, this single occurrence is not so exceptional as to support conclusions of substance.

Increased weight of the liver was present at all ages of necropsy in the enovid animals. There is no evidence that in this respect animals adapt to the treatment and the change appears to be cumulative.

The possibility of a relationship of delayed tumor onset with the slower growth of the enovid-treated rats exists, but is no more than suggested here. The decreased growth rate has been noted in all of the experiments using steroid antifertility compounds referenced. Pincus (1966) ascribed the lower body weight to inhibition of production or release of growth hormone. In either case, there is evidence of action of the compounds at the level of the central nervous system. We have come to regard such action, particularly in the hypothalamus, as relevant to cancer induction and development (Stern and Mickey, 1967; Stern, Mickey and Gorski, 1969).

### SUMMARY

A steroid antifertility combination was administered cyclically to female rats in order to simulate the intermittent schedule of oral contraception used by women.

There were alterations in endocrine and target organs which were more evident after the equivalent of 10 cycles of treatment than after 30 or 60 estrus cycles. The enovid rats also experienced an altered growth pattern.

Following a single dose of the carcinogen DMBA there was a latency in development, and modified carcinogenic potential, of induced mammary tumors. The latency was accounted for by the slower rate of growth of the enovid animals.

The modified carcinogenic response may be related to the early alteration in internal hormonal environment associated with the enovid treatment.

We thank G. D. Searle and Co. for their generous gift of Enovid E.

This study was supported by a research grant from the N.I.H., C.H.H.D.

Assistance was obtained from the Health Sciences Computing Facility—UCLA, sponsored by N.I.H. grant FR-3.

### REFERENCES

Barraclough, C. A.—(1961) *Endocrinology*, **68**, 62.

Barraclough, C. A. and Gorski, R. A.—(1961) *Endocrinology*, **68**, 68.

Blair, P. B., DeOme, K. B., and Nandi, S.—(1962) In 'Biological Interactions in Normal and Neoplastic Growth'. Edited by Brennan and Simpson. Boston (Little, Brown & Co.).

Cronkite, E. P., Shellabarger, C. J., Bond, V. P. and Lippincott, S. W.—(1960) *Radiat. Res.*, **12**, 81.

Fletcher, W. S., McSweeney, E. D. Jr, and Dunphy, J. E.—(1965) *J. Am. med. Ass.*, **191**, 116.

Gruenstein, M., Shay, H., and Shimkin, M. B.—(1964) *Cancer Res.*, **24**, 1656.

Hertz, Roy, and Bailar, J. C.—(1966) *J. Am. med. Ass.*, **198**, 136.

Holmes, R. L. and Mandl, A. M.—(1962) *J. Endocr.*, **24**, 497.
Huggins, C., Grand, L. C. and Brillantes, F. P.—(1961) *Nature, Lond.*, **189**, 204.
Huggins, C., Moon, R. C. and Morii, S.—(1962) *Proc. natn. Acad. Sci. U.S.A.*, **48**, 379.
Kahn, R. H. and Baker, B. L.—(1964) *Endocrinology*, **75**, 818.
McCarthy, D.—(1965) *Am. J. Surg.*, **110**, 720.
Minaguchi, H. and Meites, J.—(1967) *Endocrinology*, **81**, 826.
Pincus, G.—(1966) *Science, N.Y.*, **153**, 493.
Stern, E. and Mickey, M. R.—(1967) *Nature, Lond.*, **216**, 185.
Stern, E., Mickey, M. R. and Gorski, R.—(1969) *Ann. N.Y. Acad. Sci.* (In press).
Stern, E., Mickey, M. R. and Osvaldo, L.—(1965) *Nature, Lond.*, **206**, 369.—(1967) *Rass. Neurol. veg.* **XXI**, fasc. 1–2.
Weisburger, J. H., Weisburger, E. K., Griswold, D. P. Jr. and Casey, A. E.—(1968) *Life Sci.*, **7**, 259.
World Health Organization.—(1965) *Tech. Rep. Ser. Wld Hlth Org.*, **303**, 16.